# Rotavirus Gastroenteritis in a Neonatal Unit of a Greek Tertiary Hospital: Clinical Characteristics and Genotypes

**DOI:** 10.1371/journal.pone.0133891

**Published:** 2015-07-27

**Authors:** Dimitra Koukou, Panagiota Chatzichristou, Georgios Trimis, Tania Siahanidou, Anna-Venetia Skiathitou, Emmanouil I. Koutouzis, George A. Syrogiannopoulos, Athanasia Lourida, Athanasios G. Michos, Vassiliki P. Syriopoulou

**Affiliations:** 1 First Department of Pediatrics, Athens University, “Aghia Sophia” Children’s Hospital, Athens, Greece; 2 Vaccine Unit, VIANEX/Sanofi Pasteur MSD, Athens, Greece; 3 Department of Microbiology, “Aghia Sophia” Children’s Hospital, Athens, Greece; 4 Department of Pediatrics, University of Thessaly, General University Hospital of Larissa, Larissa, Greece; Laikon Hospital, GREECE

## Abstract

**Introduction:**

Rotavirus (RV) infection in neonatal age can be mild or even asymptomatic. Several studies have reported that RV is responsible for 31%-87% of pediatric nosocomial diarrhea and causes gastroenteritis outbreaks in pediatric and neonatal units.

**Objectives:**

Study clinical characteristics, genotypes and risk factors of RV infection in neonatal age.

**Methods:**

A prospective study was conducted from April 2009 till April 2013 in the neonatal special care unit of the largest tertiary pediatric hospital of Greece. Fecal samples and epidemiological data were collected from each neonate with gastrointestinal symptoms. RV antigen was detected with a rapid immunochromatography test. RV positive samples were further genotyped with RT PCR and sequencing using specific VP7 and VP4 primers.

**Results:**

Positive for RV were 126/415 samples (30.4%). Mean age of onset was 18 days. Seventy four cases (58%) were hospital acquired. Seasonality of RV infection did not differ significantly throughout the year with the exception of 4 outbreaks. Genotypes found during the study period were G4P[8] (58.7%), G1P[8] (14.7%), G12P[8] (9.3%), G3P[8] (9.3%), G12P[6] (5.3%), G9P[8] (1.3%) and G2P[4] (1.3%). RV cases presented with: diarrhea (81%), vomiting (26.2%), fever (34.9%), dehydration (28.6%), feeding intolerance (39.7%), weight loss (54%), whilst 19% of cases were asymptomatic. Comparing community with hospital acquired cases differences in clinical manifestations were found.

**Conclusions:**

Significant incidence of nosocomially transmitted RV infection in neonatal age including asymptomatic illness exists. Genotypes causing nosocomial outbreaks are not different from community strains. Circulating vaccines can be effective in prevention of nosocomial RV infection through herd immunity.

## Introduction

Rotavirus (RV) is the most common cause of viral gastroenteritis in children of preschool age worldwide [1]. In the developing countries it causes high rates of mortality and it is estimated that approximately 600000 children die each year due to dehydration from Rotavirus gastroenteritis (RVGE). In the developed countries the incidence of RV infection is also high; RV causes severe gastroenteritis and dehydration leading very often to hospitalization [2,3]. In the European countries and United States of America it is estimated that RV is responsible for 30%-50% of hospitalizations due to acute gastroenteritis among infants and young children in the prevaccine era [4,5].

Additionally, RV is the most frequent cause of viral nosocomial infections and outbreaks in neonatal units [6–10], pediatric wards [11], child care centers [9], immunocompromised patients [12] and even in aged-care facilities [13] associated with prolongation of hospitalization or rehospitalizations [1,2,4]. The first nosocomial outbreak was described in 1975 in a neonatal unit in London and since then subsequent studies described RV nosocomial infections in pediatric wards as 30%-87% of nosocomial diarrhea cases. In neonatal units the prevalence rate of RV is 1.4%-56% [14–16] and it is rather underestimated as the clinical presentation of the infection in this age is mild or even asymptomatic [17]. There are studies, though, that relate neonatal RV infection to more serious symptoms such as bloody-mucous stools, abdominal distension and necrotizing enterocolitis (NEC) [18–20]. In older ages, especially in the age of 3 months to 3 years, the infection is often serious and causes symptoms such as diarrhea, vomiting and fever, dehydration, abnormal electrolyte values and even death. The first episode of gastroenteritis is always the most severe, whereas the following are milder.

RV seasonal distribution in the countries with temperate climate occurs mainly during late winter and spring months although there is divergence from year to year because RV gastroenteritis follows an annual pattern. Usually, in Europe, the season begins from south-western countries in the beginning of winter and expands to north-eastern countries at the end of spring [21]. RV nosocomial outbreaks however can happen at any time of year and usually occur during months there is high incidence of RV in community [2].

There are few reports on the symptoms associated with RV infections in neonates. Previous studies have reported that diarrhea is less common in neonates than in older infants and most neonates are asymptomatic. Neonates are susceptible to RV even in their first days of life. Immaturity of intestine, lack of genotype specific antibodies and the unconfirmed protection of maternal antibodies are predisposing factors that are mentioned in the literature [16]. Risk factors that are associated with RV nosocomial infection in neonatal units are not easy to be determined and generally few factors have been mentioned in published studies; low birth weight, prematurity, non-breastfeeding and underlying disease. However the real factors that are responsible for the large outbreaks in hospital wards appear to be the extensive viral contamination due to the low infective dose and the susceptibility of the virus against the usual disinfectants that make outbreaks difficult to control despite hygiene measures [2].

In Greece, there are few epidemiological studies describing RVGE in children up to the age of 5 years and there is uncertainty about the presence and severity of clinical signs associated with RV in neonates. The prevalence of RVGE in hospitalized children <5 years of age with acute gastroenteritis has a range between 23.8% and 48% [22–24], while the prevalence of RV in nosocomially transmitted infections in Greek hospital units is 21.3% [22]. The RV infection in the neonatal age has not been studied extensively. Studies are published to date are few, planned differently and have a lot of restrictions. The aim of this study was to describe the epidemiology of RV infection in the neonatal age and to estimate the incidence of nosocomial infection in hospitalized neonates in Greece.

## Material and Methods

A prospective study was carried out from 1st April 2009 up to 31st March 2013 in the neonatal unit of the “Aghia Sophia” Children’s University Hospital, which is the largest tertiary hospital situated in Athens, capital of the prefecture of Attica. Neonates are referred and admitted to the hospital by private pediatricians of the community or by maternity hospitals; neonates presenting symptoms of respiratory infectious diseases or gastrointestinal infectious diseases are hospitalized in different wards.

During the study all admitted neonates were screened daily for gastrointestinal symptoms, such as diarrhea with or without blood or mucous, increased number of stool (≥3/day) and/or vomiting. Stool sample was collected from each neonate with one or more gastrointestinal symptoms or from neonates without symptoms, who were simultaneously hospitalized in the same room with a RV case. Stool samples were kept in 4°C and tested within 24 hours for RV antigen with a rapid immunochromatographic test (VIKIA test, *Biomerieux*) that detects Rota antigen with high sensitivity and specificity [25]. After the test, samples were separated into two categories; RV positive samples and RV negative samples. The RV positive samples, that were available and properly stored, were further G and P typed with multiple nested PCR using specific primers for VP7 and VP4 genes respectively, according to the published protocol of the European Rotavirus Network [21]. RV genotypes in neonatal age were defined and genotype distribution was classified into different RV seasons. Every RV season was considered starting in September and ending in next August.

Additionally, in each neonate with RV positive sample, epidemiological and clinical data were recorded including gender, origin, place of stay, gestational and postnatal age, type and weight of birth, type of feeding, duration of hospitalization, clinical characteristics of gastroenteritis (change in stool constitution, vomiting, fever), complications (dehydration, loss of weight, seizures, intussusception, necrotizing enterocolitis [NEC], septicemia), co-infection, type and duration of treatment and laboratory data. Fever was defined as axillary temperature ≥37.5°C. Finally the positive for RV samples cases were further separated in hospital acquired (HA) and community acquired (CA) and analyzed statistically. Hospital acquired infection was defined as the presence of RV in stool sample of neonates at least 72 hours after admission in the hospital, provided that previously no symptoms of gastroenteritis were presented. Outbreak was defined as a cluster of 5 or more cases of rotavirus diarrhea per week.

The study was approved by the Ethics Committee of “Aghia Sophia” Children’s Hospital, including consent procedure; a written informed consent was obtained from each child’s parent or legal guardian. The data were analyzed anonymously. The statistical analysis of data was performed by using SPSS version 19.0 (IBM inc.). The qualitative data were analyzed by Fisher-exact test, while the quantitative data by Student’s t-test or Mann-Whitney test, as appropriate. A confidence level of 5% was considered statistically significant.

## Results

From the total of 1241 neonatal admissions recorded at the period between April 2009 and April 2013, stool sample was collected from 415 neonates who were tested for the antigen of RV. Positive for RV were 126/415 samples (30.4%). Male were 69/126 (54.8%). Rest of the neonates was not included in the study, because either they did not present symptoms of gastroenteritis, not were simultaneously hospitalized in the same room with a RV case, or because parents did not consent to give stool sample for testing.

The mean age of neonates at the day of sample collection was 18 days (range 2 to 30 days). Premature (gestational age <37 weeks) were only 7.9% (10 neonates) and low birth weight (<2500 g) were 7.1% (9 neonates). The mean duration of hospitalization was 17 days and the mean time for the diagnosis of RV from the day of admission was 6 days (range; 0 to 86 days). Nosocomial infection was the cause of RVGE in 58% of samples. The demographic characteristics of neonates tested are presented in Table 1.

**Table 1:** Demographic characteristics of Rotavirus positive cases and comparison of Hospital acquired (HA) and Community acquired (CA) cases.

Demographics	Total	HA	CA	P Value
	n = 126 (%)	n = 74 (%)	n = 52 (%)	
Gender				
Male	69 (54.8)	43 (58.1)	26 (50.0)	0.23
Female	57 (45.2)	31 (41.9)	26 (50.0)	
Birth weight (g)				
Low birth weight ≤2500 g	9 (7.1)	7 (9.5)	2 (3.8)	0.19
Normal birth weight >2500 g	117 (92.9)	67 (90.5)	50 (96.2)	
Gestational age (wk)				
Full-term ≥37	116 (92.1)	68 (91.9)	48 (92.3)	0.60
Premature <37	10 (7.9)	6 (8.1)	4 (7.7)	
Labour				
Normal delivery	64 (50.8)	36 (48.6)	28 (53.8)	0.34
Cesarean section	62 (49.2)	38 (51.4)	24 (46.2)	
Type of feeding				
Only breast milk	29 (23.0)	10 (13.5)	19 (36.5)	0.001
Formula and breast milk	43 (34.1)	23 (31.1)	20 (38.5)	
Only formula	54 (42.9)	41 (55.4)	13 (25.0)	
Origin				
Greek	61 (48.4)	38 (51.4)	23 (44.2)	0.55
Roma	13 (10.3)	6 (8.1)	7 (13.5)	
Foreigner	52 (41.3)	30 (40.5)	22 (42.3)	
Region				
Urban	100 (79.4)	58 (78.4)	42 (80.8)	0.46
Rural	26 (20.6)	16 (21.6)	10 (19.2)	
Season				
Winter	27 (21.4)	13 (17.6)	14 (26.9)	0.14
Spring	27 (21.4)	15 (20.3)	12 (23.1)	
Summer	38 (30.2)	28 (37.8)	10 (19.2)	
Autumn	34 (27.0)	18 (24.3)	16 (30.8)	

**Fig 1:**
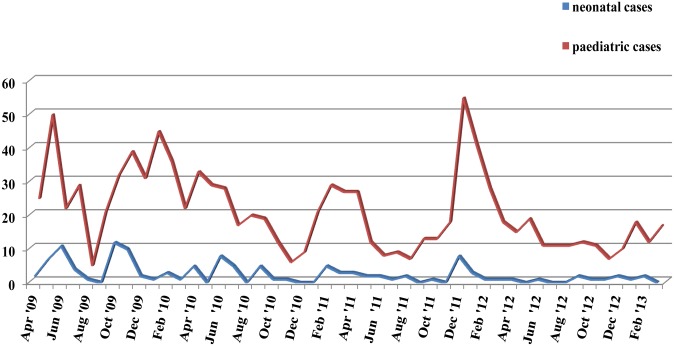
Seasonal distribution of RV cases in hospitalized pediatric patients and neonates.

The seasonal distribution of RV positive samples in the study population showed that RV infection had increased prevalence during summer (30.2%) and autumn (27%). Comparatively with the seasonal distribution of RV positive samples of children of preschool age that were hospitalized due to gastroenteritis during the same period and in the same hospital, it appears that small outbreaks in the neonatal unit occurred simultaneously or after periods with high incidence of RV in the community (Fig 1).

In neonates positive for RV, the most frequent reasons for hospitalization were fever (29.4%), diarrhea (15.1%), nonspecific symptoms; feeding intolerance, consciousness disturbance (11.9%), respiratory symptoms (9.5%) and vomiting (7.9%) (Table 2). The clinical characteristics of neonates positive for RV and the comparison between HA and CA cases are also shown in Table 2. Changes in the consistency and/or the number of stool were recorded in 81% of cases; watery stool in 58.7% of neonates, mix with mucous in 35.7% and mix with blood in 16.7%. Vomiting was presented in 26.2% of neonates and fever in 34.9%; abdominal distention in 15.1% and feeding intolerance in 39.7%; weight loss more than 3% of body weight in 54% of neonates and other signs of mild or severe dehydration in 28.6% of them. Altered level of consciousness was observed in 11.1% of neonates, whereas 12.7% were seriously ill with unstable vital signs (tachycardia or tachypnea) and 7.9% had septicemia. Co-infection, viral or microbial was found in 50% of neonates; RSV respiratory infection and urinary tract infection (*E*.*coli*, *Klebsiella pneumoniae*) were the most frequent co-infections, respectively. Laboratory testing in stool samples for co-infection showed adenovirus in 3 samples and Salmonella and Campylobacter in 2 samples. No neonate positive for RV presented complications from CNS (seizures) or intussusception. Finally, 7 neonates presented NEC of stage I by Bell’s scale (fever, unstable vital signs, lethargy, abdominal distention, bloody stool).

**Table 2:** Clinical characteristics of Rotavirus positive cases and comparison of Hospital acquired (HA) and Community acquired (CA) cases.

Clinical manifestations	Total	HA	CA	P Value
	n = 126 (%)	n = 74 (%)	n = 52 (%)	
**Clinical manifestations on admission**				
Fever	37 (29.4)	20 (27.0)	17 (32.7)	<0.001
Diarrhea	19 (15.1)	1 (1.4)	18 (34.6)	<0.001
Feeding intolerance/ Disturbance of consciousness	15 (11.9)	13 (17.6)	2 (3.8)	<0.001
Respiratory symptoms	12 (9.5)	9 (12.2)	3 (5.8)	<0.001
Vomiting	10 (7.9)	2 (2.7)	8 (15.4)	<0.001
Jaundice	5 (4.0)	4 (5.4)	1 (1.9)	<0.001
Seizures or tremor	5 (4.0)	5 (6.8)	0 (0.0)	<0.001
Prematurity	2 (1.6)	2 (2.7)	0 (0.0)	<0.001
**Clinical features of RV infection during hospitalization**				
Diarrhea	102 (81.0)	60 (81.1)	42 (80.8)	0.57
Vomiting	33 (26.2)	15 (20.3)	18 (34.6)	0.05
Fever	44 (34.9)	26 (35.1)	18 (34.6)	0.55
Consistency of stool				
watery	74 (58.7)	48 (64.9)	26 (50.0)	0.06
mucoid	45 (35.7)	20 (27.0)	25 (48.1)	0.04
bloody	21 (16.7)	8 (10.8)	13 (25.0)	0.06
Abdominal distention	19 (15.1)	6 (8.1)	13 (25.0)	0.01
Feeding intolerance	50 (39.7)	30 (40.5)	20 (38.5)	0.45
Weight loss	68 (54.0)	48 (64.9)	20 (38.5)	0.007
Vital signs unstable	16 (12.7)	7 (9.5)	9 (17.3)	0.15
Altered level of consciousness	14 (11.1)	2 (2.7)	12 (23.1)	0.001
NEC	7 (5.6)	2 (2.7)	5 (9.6)	0.10
Septicaemia	10 (7.9)	6 (8.1)	4 (7.7)	0.63
Dehydration				
≤5%	31 (24.6)	15 (20.3)	16 (30.8)	0.23
>6%	5 (4.0)	2 (2.7)	3 (5.8)	0.23
Asymptomatic	24 (19.0)	14 (18.9)	10 (19.2)	0.57
**Other variables**				
Co-infection	63 (50.0)	42 (56.8)	21 (40.4)	0.05
Positive stool culture	2 (1.6)	1 (1.4)	1 (1.9)	0.65
Antibiotic treatment	103 (81.7)	59 (79.7)	44 (84.6)	0.32

Statistically significant differences (p<0.05) between HA and CA RV positive cases were detected as regards the type of feeding; HA cases were formula fed, the consistency of fecal samples; CA had mixture of mucous and blood in the sample, the presence of clinical symptoms; CA presented more often with vomiting, abdominal distention and altered level of consciousness (lethargy or hypotonia), whilst HA had more often watery stool and significant weight loss. RV positive neonates with no gastrointestinal symptoms (changes in number or/and consistency of stool or/and vomiting) were 24 (19%). Comparison of quantitative data between HA and CA cases is presented in Table 3. HA cases were of older age and lower birth weight than CA and had longer duration of hospitalization. No difference in the incidence of prematurity between HA and CA cases was observed. Similar numbers of vomiting and diarrhea episodes were recorded in HA and CA cases but longer duration of vomiting was presented in the latter group. As regards the laboratory data, RV infection presented with normal white blood cell count, normal or mildly elevated serum C-reactive protein levels and no abnormal electrolytes or changes in acid-base balance (Table 3).

**Table 3:** Comparison of quantitative data in Hospital acquired (HA) and Community acquired (CA) Rotavirus positive cases.

Variable	HA±SD (n = 74)	CA±SD (n = 52)	P Value
Age (days)	19.9±7.3	16.2±7.5	0.009
Hospitalization days	17.5±13.0	11.3±8.6	0.005
Gestational age (weeks)	37.9±1.8	38.3±1.2	0.07
Birth weight (g)	3051.8±548.2	3264.8±465.4	0.02
Diarrhea per 24h (n)	3.9±2.3	3,3±1.4	0.16
Duration of diarrhea (days)	2.5±2.4	2.3±1.8	0.81
Vomiting per 24h (n)	1.8±1.2	2.4±1.3	0.19
Duration of vomiting (days)	1.3±0.5	2.4±1.5	0.02
Duration of fever (days)	1.6±1.2	1.6±1.0	0.98
WBCs x 10^3^cells/μL	9.46±4.96	11.69±4.80	0.01
Hb (g/dl)	11.1±2.9	13.4±2.3	<0.001
PLTs x 10^3^cells/μL	426.75±165.03	416.81±135.92	0.72
CRP (mg/L)	30.9±19.8	27.4±18.1	0.70
Glucose (mg/dl)	85.3±18.8	88.2±20.4	0.41
Urea (mg/dl)	15.7±8.2	16.4±6.2	0.61
Creatinine (mg/dl)	0.37±0.1	0.34±0.2	0.41
AST (IU/L)	37.3±10.5	36.9±16.1	0.85
ALT (IU/L)	25.9±9.3	20.9±8.9	0.003
γGT (IU/L)	90.4±74.9	87.5±47.8	0.81
Bilirubin total (mg/dl)	5.1±4.2	6.0±4.8	0.38
Potassium (mmol/L)	4.8±0.6	5.0±0.6	0.01
Sodium (mmol/L)	137.9±2.3	137.6±2.2	0.50
Chloride (mmol/L)	103.9±3.2	103.0±2.2	0.11

WBCs, White Blood Cells; Hb, Hemoglobulin; PLTs, Platelets; CRP, C-Reactive Protein; AST, Aspartate Transaminase; ALT, Alanine Transaminase; γGT, Gamma Glutamyl Transferase.

**Fig 2:**
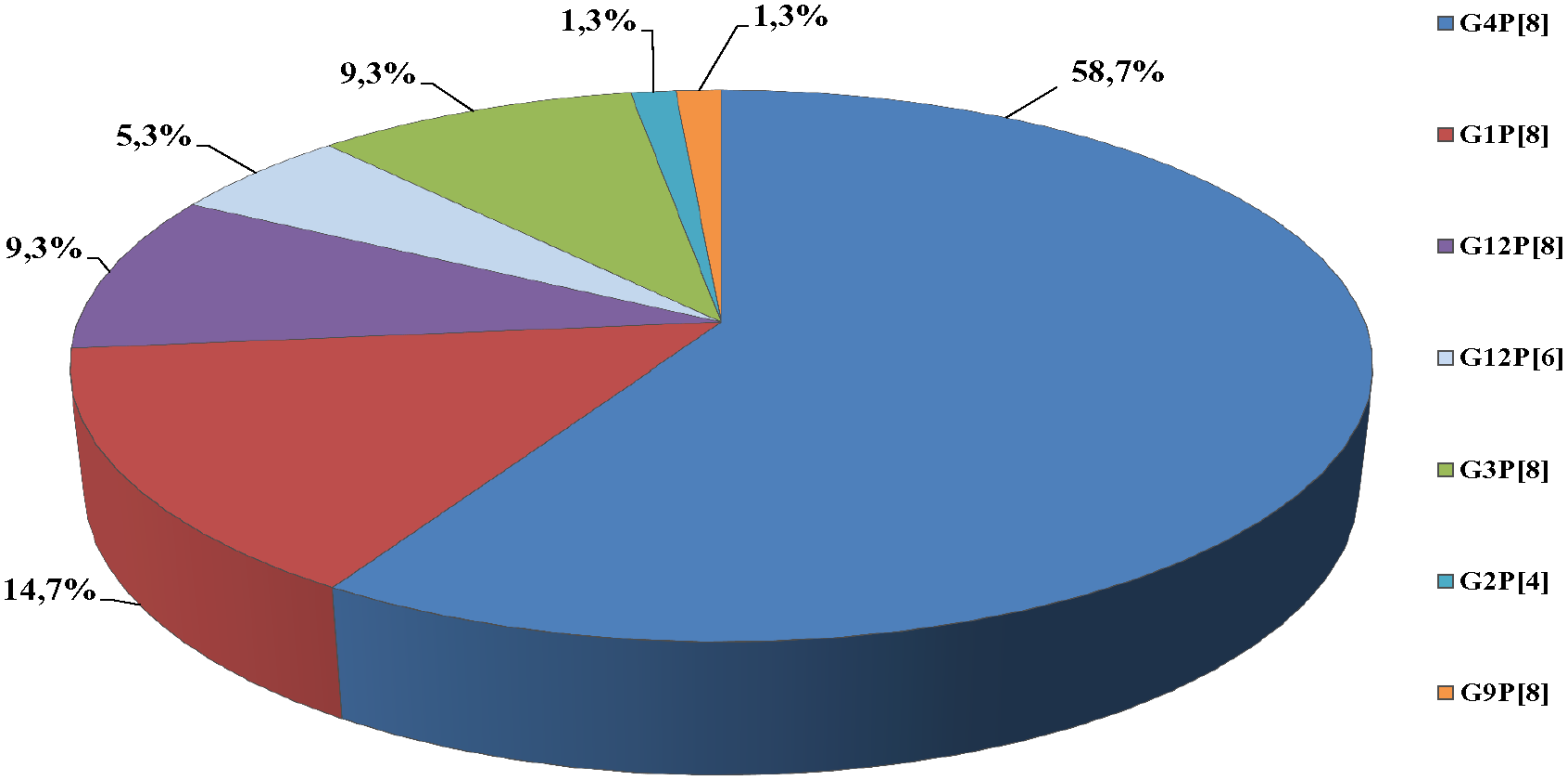
RV Genotypes distribution in neonatal cases during the 4-year study period (n = 126).

**Fig 3:**
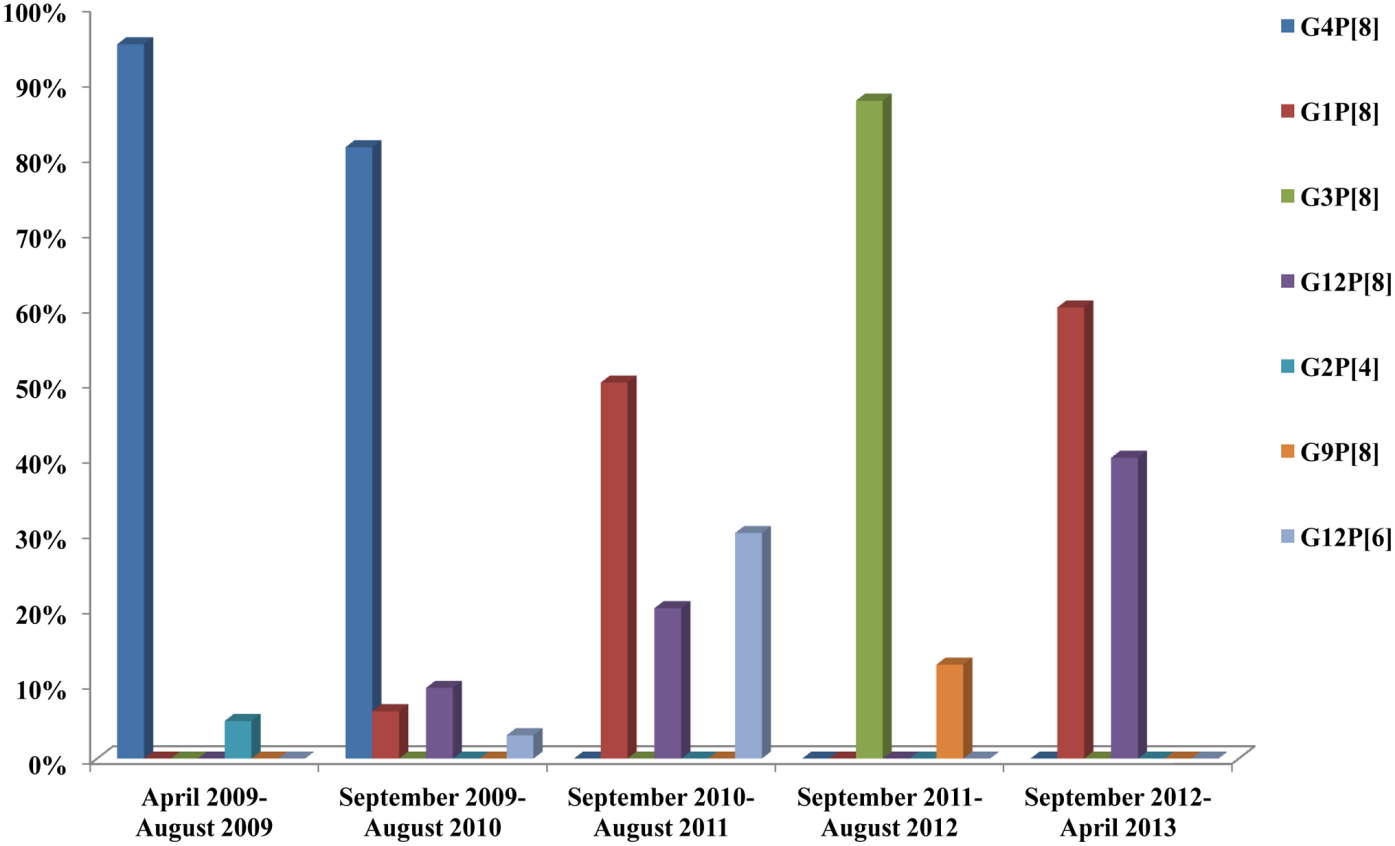
Annual distribution of RV in neonatal cases (n = 126).

Genotypes detected from RV positive samples were as follows: G4P[8] (58.7%), G1P[8] (14.7%), G12P[8] (9.3%), G3P[8] (9.3%), G12P[6] (5.3%), G9P[8] (1.3%) and G2P[4] (1.3%) (Fig 2). A statistically significant correlation between season and genotypes distribution was observed. Specifically G4P[8] was more frequent during summer, spring and autumn, while G3P[8] and G1P[8] were more frequent during winter (p<0.001). Moreover, genotypes distribution differed from year to year (p<0.001) (Fig 3), whereas it did not differ significantly between community and hospital acquired strains or between male and female.

## Discussion

The present study is the first reported in Greece as regards the epidemiology and clinical characteristics of RV infection in the special group of neonates; data were collected from the largest Greek pediatric hospital. The prevalence of RV infection (30.4% of admissions due to acute gastroenteritis) is within the range reported in previous studies (1.7%-50%) but it is considered high, because comparatively with previous studies, that were carried out in neonatal population for a specific period of year, the present study was conducted during the whole year and also for 4 years consecutively. The high RV incidence can be attributed to the fact that samples were collected mostly from neonates that were suspect for RV infection and also from asymptomatic neonates, who could be subclinical carriers of the virus. The surveillance was limited to the hospitalization period, so infections that were hospital acquired and developed after discharge were ignored; recognition of these cases could lead to an even higher incidence of RV infection, as other studies have shown (i.e. Gianino et al showed that 67% of symptomatic RV cases were recognized after discharge) [26].

Among neonates, RV infection is believed to be less common and more often asymptomatic [27–29]. Although the existence of asymptomatic RV infection is a fact, it is rarely reported in the literature leading to an underestimation of its incidence. Asymptomatic RV infection was found in a percentage of 19%, which agrees with other studies in which the asymptomatic infection is estimated between 11% and 39% [6, 26].

Most cases (58%) of RVGE in neonates were hospital acquired. Tai Chen et al [8] recognized 72.4% of RVGE cases in neonates as hospital acquired, while Shim Jung Ok et al [6] reported a rate of 93% for nosocomial infection in preterm infants. Further statistical analysis comparing nosocomial with community samples was carried out, so that differences in the clinical picture of illness to be found.

Clinical manifestation of RV infection in neonates has been associated with diarrhea (64%-77%) [30,31] presenting with watery stool in term neonates and bloody mucoid stool in preterm neonates [32]. Severe clinical symptoms like bloody diarrhea associated with abdominal distention resulting in NEC and even bowel perforation has been reported, especially in preterm and low birth weight neonates hospitalized in special care units, whilst vomiting, weight loss and fever has been rarely described in neonatal age [20,31,33]. In this study the clinical picture of RV infection included mostly change in the consistency of stool (mixture with mucous and blood), vomiting, abdominal distention and altered level of consciousness. These symptoms were statistically correlated with CA RVGE. In contrast, patients with HA RV infection presented more often with watery stool and significant weight loss.

It has been reported that maturational changes in intestine of premature neonates lead to increased incidence of NEC after RV infection [34,35]. This can be an explanation why preterm neonates present different symptoms in comparison with full term neonates or infants of older age; instead of fever and watery stool, preterm infants present blood/mucous in stool and NEC when RV infection occurs [8,32,33]. This study was carried out in a neonatal unit where neonates are referred by obstetric clinics and pediatricians in private practice and the percentage of premature or low birth weight was very low. Thus, in comparison with previous studies, the age and the birth weight of hospitalized neonates was higher, resulting in decreased prevalence of NEC (5.6%).

The seasonal distribution of RV in neonates appears to be less specific than in older ages where there is a winter and spring peak of RVGE in temperate climates. In the present study small outbreaks were marked mainly during the months of summer and autumn, which are periods that hospital’s personnel is limited in number and measures of prevention of transmission of nosocomial infections are not always strictly applied because of the large load of work versus the small number of personnel. Moreover, outbreaks in the neonatal unit were marked after RV epidemics in community settings. The outbreaks were limited due to the Unit infection control policies and practices. Focus was set to both isolation of infected neonates and more vigorous surveillance and implementation of control measures. Staff was trained in infection control policies and practices according to relative guidelines of Hospital Infection Committee. Comparing data between neonates that were nosocomial infected and neonates with community acquired RV infection, risk factors for the nosocomial transmission of the virus were not found. In general it is difficult to determine risk factors for the transmission of RV. It appears that the high contagiousness via fomites, the inadequate sterilization of surfaces and hands with the usual measures of hygiene, as well as the subclinical transmission to the hospital’s personnel, play significant role for the spread of virus and the occurrence of small nosocomial outbreaks. Conflicting data regarding the role of breastfeeding as a protective factor against RV infection exist. Some authors report that breastfeeding is a protective factor especially in newborns [36,37,38–41], other report that breastfeeding protects only from severe disease, whereas other studies have shown that there is no difference in frequency of RV between breastfed and formula fed infants [37,42,43]. Widdowson et al [44] reported that high levels of RV antibodies in mothers may not protect infants and the environment may be the most important reservoir of RV during outbreaks. Generally longer hospital stay, that correlates with smaller and sicker neonates, increases the likelihood of nosocomial RV transmission [29,33].

A lot of studies have shown that genotypes circulating in neonatal nurseries are distinct from genotypes circulating in community [14,45,46]. There are various studies that describe neonatal epidemics from strains containing the P[6] gene in combination with the G1, G2, G3, G4 and G9 like G2P[6], G4P[6] and G9P[6] [6,7,15,16,27,47,48], consisting strains that are possibly animal originated. Similar strains have caused outbreaks in India [49], South Africa [50] and Brazil [51,52]. Additionally, in India, several neonatal outbreaks were attributed to P[11] bearing either G3, G9 or G10 specificity [47,48,53]. For the first time in 1994, Das et al [54] published that genotypes isolated from neonates in New Delchi (India) during the period 1986–1992 (G9P[11], G9P[6], G3P[8] and G2P[4]) were not only animal originated but also common human originated. Recently it was demonstrated by phylogenetic analysis that strains containing P[6], which cause outbreaks in infants, can also cause epidemics in the community [55].

The present study included not only hospital acquired but also community acquired RV infections and seven genotypes were isolated; common human originated and uncommon possibly animal originated. Genotypes differed by year of isolation. Thus, from the beginning of the study up to September 2010 the most common isolated genotype was G4P[8]; the next months up to September 2011, the isolated genotypes were G1P[8], G12P[8], G12P[6]; up to September 2012, the G3P[8] and G9P[8] were detected, whereas up to April 2013, the G1P[8] and G12P[8] were found. A high percentage of genotypes detected (85%) were similar with the 5 most frequent genotypes circulating around the world and in the community; G1P[8], G2P[4], G3P[8], G4P[8] and G9P[8]. The remaining genotypes (15%) were combination of the possibly animal originated G12 genotype with the common P[8] or the animal originated P[6] genotype. No significant difference was found between CA RV and HA RV genotypes, as RV infection in the neonatal unit can be influenced by RV strains of the community. A dominant strain was isolated during each of the four outbreaks (May 2009, October 2009, June 2010, December 2011); G4P[8] in the first three outbreaks and G3P[8] in the last one, similar to the genotypes isolated from children hospitalized with RV gastroenteritis in the same hospital and the same period.

Available vaccines can be effective in prevention of nosocomial neonatal RV infection through herd immunity, as the first dose cannot be administered earlier than 6 weeks of age. RV vaccines are available in Greek private market since 2007. Vaccination coverage was estimated close to 25% from 2008 to 2011. Following optional recommendation, since the beginning of 2012, coverage reached 35%. Differences in RV genotypes isolated annually it is more likely to be attributed to the natural seasonal genotype fluctuation than to the vaccine implementation, as the vaccine coverage is relatively low and especially if it is considered that no strain different from those included in the vaccines was prevalent in our study. A study modeling the impact of universal Rotavirus vaccination in Greece showed the potential reduction of nosocomial Rotavirus infections [56].

Limitations of our study are the small number of sample (126 patients), the absence of an objective system of recording nosocomial infections and the subjective assessment, by a medical doctor, of the presence of diarrhea. Consequently, our results cannot represent the whole population of neonates. Nevertheless, our study is the first one in Greece that describes the epidemiology of RV in hospitalized neonatal population.

In conclusion the results of this study show that RV infection in hospitalized neonates is nosocomially transmitted in a high percentage (58%). The infection in neonatal age can present subclinically in almost 19% of cases or with change in stool consistency and non-specific symptoms, such as consciousness disturbance and feeding intolerance as well as with vomiting and fever. Asymptomatic neonatal cases, hospital personnel and environment (surfaces, medical tools) play a role as reservoir for RV and in combination with the high viral contamination can lead to outbreaks in neonatal units. Reported risk factors, such as prematurity and type of feeding, are not confirmed by this study. Genotypes isolated in neonatal units cause small outbreaks and can be similar with those strains circulating in children of older ages in community or in hospitalized children. It is important more studies to be conducted so as the picture of RVGE in neonatal age to be elucidated.
